# Per-endoscopic rectal fistulotomy: A new endoscopic rectal myotomy technique to support the management of complex anorectal fistulas

**DOI:** 10.1055/a-2924-5684

**Published:** 2026-08-21

**Authors:** Timothee Wallenhorst, Maria Parmigiani, Amandine Landemaine, Fabien Pinard, Thomas Grainville, Charlène Brochard, Laurent Siproudhis

**Affiliations:** 1 Department of Endoscopy and Gastroenterology 36684 Rennes University HospitalRennesBrittanyFrance; 2 Department of Translational Medicine and Surgery 60234 Università Cattolica del Sacro Cuore Facoltà di Medicina e ChirurgiaRomeLazioItaly; 3 Department of Gastroenterology and Endoscopy 188700 Fondazione Poliambulanza Istituto OspedalieroBresciaItaly; 4 Gastroenterology and Endoscopic Unit 55151 Hospital Center CornouailleQuimperBrittanyFrance

Intramural anorectal fistulas are characterized by the presence of one or several fistulous tracts, which may diffuse into the upper pelvirectal space above the levator ani muscle. They are difficult to manage, with a high risk of postoperative recurrence. 1​

We report a new technique called “PERF: Per-Endoscopic Rectal Fistulotomy” for the treatment of anorectal suppuration refractory to conventional surgical treatment when it has not been possible to guide the fistula through a seton.

In our case, a 66-year-old man developed a complex supra-levator anorectal suppuration from an intra-mural abscess, diffusing above the upper pelvic space. This chronic fistula had been evolving for 30 months, refractory to three conventional transanal fistulotomy surgeries and responsible for daily anal purulent discharges. Magnetic resonance imaging (MRI) showed a path of chronic suppuration, measuring 8 mm thick and 60 mm long, developed outside the internal circular rectal muscle layer, in the shape of a horseshoe.

**Fig. 1:**
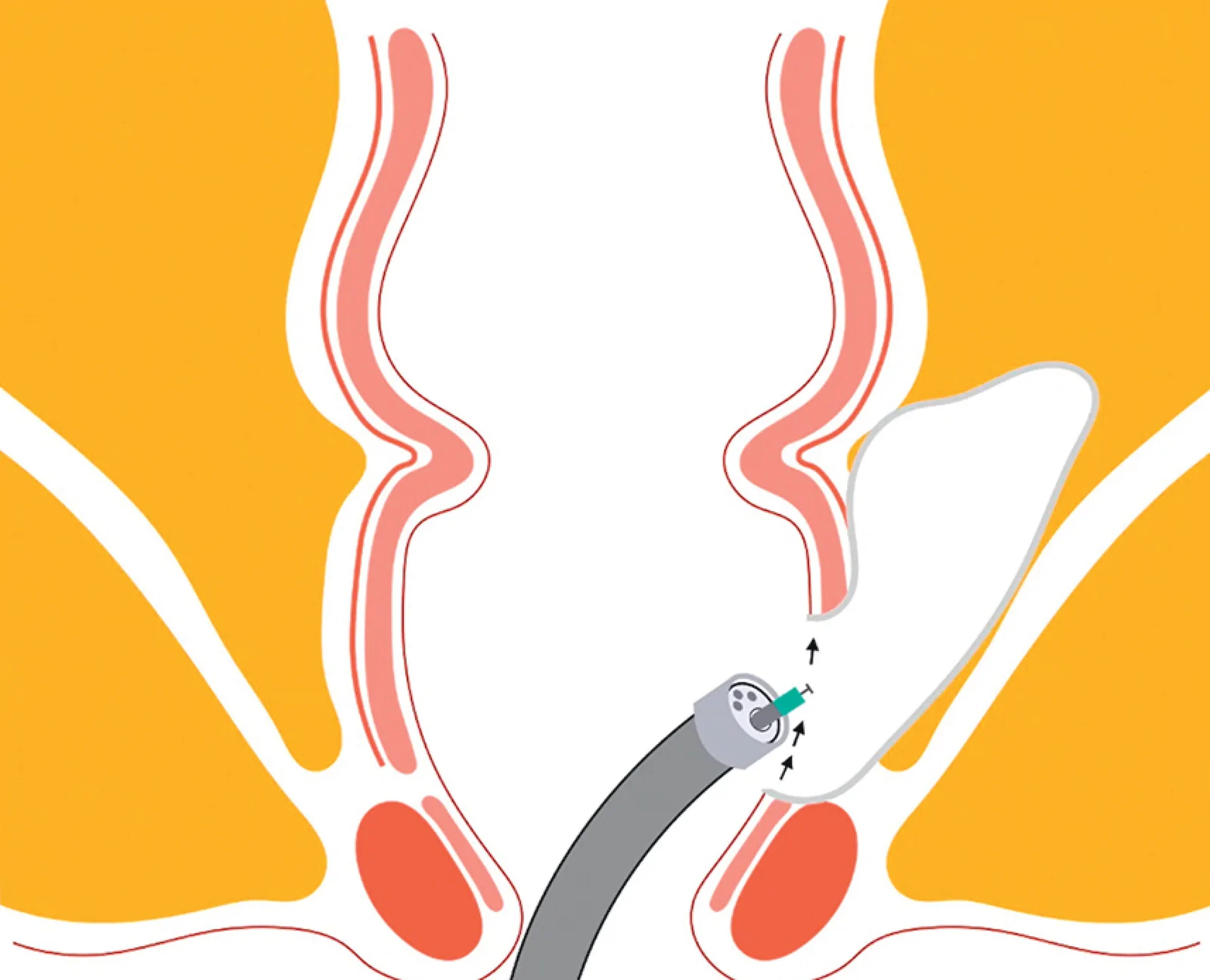
Schematic representation of progressive myotomy to the top of the collection.

As shown in Video 1 , the procedure involves the following steps: Step 1: The fistulous orifice is identified using a gastroscope. Step 2: Catheterization of the fistulous tract with a guide wire. Step 3: Mucosal incision starting from the orifice towards the top of the collection with Flushknife BT 1.5 (Fujifilm, Japan). The submucosal plan was not identified due to intense fibrosis. Step 4: Myotomy of the internal circular muscular layer progressively until the granulation tissue is reached, which serves as the landmark to define the depth of the myotomy ( Fig. 1 ). Step 5: Complete opening of the suppuration from the anal part to the oral part into the rectal lumen. Step 6: Granulation tissue is debrided with hot forceps to clean the bottom of the suppuration, then electrocoagulated in forced coagulation mode to stimulate tissue regeneration and promote fistula healing.

**Video 1** Per-endoscopic rectal fistulotomy: a new endoscopic rectal myotomy technique to support the management of complex anorectal fistulas. Video-URI:


The success of the PERF procedure is defined by the complete regression of anal purulent discharge symptoms and disappearance of suppuration on MRI at 6 months, which was the case here. The long term follow-up at 12 months showed no recurrence.

This new endoscopic rectal myotomy technique “PERF” represents an alternative treatment option for intramural supra-levator fistulas refractory to conventional surgery.

Endoscopy_UCTN_Code_TTT_1AQ_2AG
